# Hypertensive Hypoalgesia in a Complex Chronic Disease Population

**DOI:** 10.3390/jcm10173816

**Published:** 2021-08-25

**Authors:** Meaghan Ferguson, Maxwell Slepian, Christopher France, Anton Svendrovski, Joel Katz

**Affiliations:** 1 Department of Psychology, York University, Toronto, ON M3J 1P3, Canada; 2 Department of Anesthesia and Pain Management, Toronto General Hospital, Toronto, ON M3J 1P3, Canada; Maxwell.slepian@uhn.ca; 3 Department of Psychology, Ohio University, Athens, OH 45701, USA; france@ohio.edu; 4 UZIK Consulting Inc., Toronto, ON M5B 2J1, Canada; anton.toronto@gmail.com

**Keywords:** health, pain, hypertension, hypoalgesia, chronic, disease

## Abstract

Hypertension-related hypoalgesia, defined as lower pain sensitivity in individuals with high blood pressure, has yet to be examined in a large-scale study of complex care residents. Here, the Continuing Care Reporting System database, which contains health information on residents of Canadian complex chronic care facilities, was used for assessment. Hypertension was reported among 77,323 residents (55.5%, total *N* = 139,920). Propensity score matching, with a 1:1 ratio, was used to identify a control record without hypertension for each case. Multinomial logistic regression was used to quantify the effects of hypertension and sex on four-level ordinal pain variables, controlling for potential confounders. The matched dataset included *n* = 40,799 cases with hypertension and *n* = 40,799 without hypertension, with 57% female. Residents with hypertension had significantly lower odds of reporting pain (yes/no) (*OR* = 0.85, 95% CI 0.81–0.90, *p* < 0.001), including on measures of severe pain (*OR* = 0.69, 95% CI 0.63–0.76, *p* < 0.001). A significant interaction between hypertension and sex (*OR* = 1.17, 95% CI 1.03–1.32, *p* = 0.014) indicated that a significantly greater proportion of females without hypertension reported severe pain (8.71%). The results confirm the relationship between hypertension and reduced pain sensitivity on a population level.

## 1. Introduction

Hypertension is a highly prevalent chronic condition among adults and is associated with a high rate of multimorbidity [1]. Elevated blood pressure (BP) affects 40% of the global adult population aged ≥25 years, which corresponds to more than 1 billion individuals worldwide (World Health Organization, 2013). In a population of individuals with complex chronic diseases, hypertension is often comorbid with other cardiovascular diseases (CVDs; e.g., coronary heart disease, stroke, congestive heart failure, peripheral vascular disease, and end-stage renal disease), diabetes, depression, anxiety, and acute and chronic pain [2,3]. In conjunction with hypertension, acute and chronic pain are also highly prevalent conditions that affect wellbeing and quality of life [4].

Evolving research investigating the relationship between hypertension and pain suggests that hypertension is associated with reduced pain sensitivity, a phenomenon known as hypertensive hypoalgesia [5,6,7,8,9,10,11,12,13,14,15,16]. Epidemiological and meta-analytic studies support the notion of reduced pain sensitivity in humans and decreased nociceptive responsiveness in laboratory animals with hypertension as compared to those with normotensive blood pressure levels [17,18,19,20]. Further, there is emerging evidence to suggest that decreased pain perception may result from a common physiological dysfunction, suggesting that hypoalgesia may actually precede the onset of hypertension and may be a behavioural marker or risk factor for hypertension [18,21,22,23,24,25].

Particularly in the context of an aging population with multiple comorbidities, a better understanding of the ways diseases interact is critical to enhancing patient management and the growing demands on the healthcare system. With a few exceptions, hypertension-related hypoalgesia has generally been studied in small samples, but to date no study has examined this relationship using a large database of complex care residents, despite the prevalence of these common, often comorbid, health issues in such settings. Accordingly, the aim of the current study was to examine the association between hypertension and pain within a large complex chronic disease (CCD) population. Based on the literature reviewed above, we hypothesized that pain would be less prevalent and less severe among residents with hypertension than in a propensity-matched control sample without hypertension.

## 2. Materials and Methods

Data source. This study used data from the Canadian Institute for Health Information (CIHI) Facility-Based Continuing Care Reporting System (CCRS), which captures information on individuals admitted to publicly-funded hospital facilities for complex chronic care in Canada (Continuing Care Reporting System, 2013–2014) between the years 2006–2016. Upon entry to a complex chronic care (CCC) facility, residents are administered a standardized assessment protocol, the Resident Assessment Instrument—Minimum Data Set/Full Assessment (RAI/MDS/FA 2.0 Canadian Version), within 14 days of admission. If the resident remains in the facility for longer than 92 days, quarterly assessments are conducted. These assessments are typically completed by the treating nurse or physician and include resident self-reports and information from medical files. Residents from Ontario-based facilities between the ages of 18 and 101 years, assessed between the years 2006 and 2016, were included in the study.

The RAI/MDS/FA 2.0 is an internationally validated clinical assessment instrument [26,27,28,29,30,31]. This standard tool collects a wide array of information, including basic demographic characteristics, diagnostic profiles, medication usage and treatment participation, and outcomes, and also categorizes diseases.

Ethical approval for this study was obtained through the CIHI ethics review board, July 2020.

Hypertension Diagnosis and Propensity Score Matching (PSM). The original full CCRS dataset consisted of 139,920 residents, of whom 77,323 had a diagnosis of hypertension. The diagnosis of hypertension was taken directly from the CCRS RAI/MDS/FA 2.0. Propensity score matching was used to create matched pairs of residents: cases (with hypertension) and controls (without hypertension), using a 1:1 ratio and ensuring the case and control residents had the exact same values for age, gender, marital status, assessment year and total disease count.

Pain measures. The CCRS RAI/MDS/FA 2.0 measures pain using two descriptive scales:

1. Frequency: no pain (0), pain less than daily (1), pain daily (2);
2. Intensity: mild pain (1), moderate pain (2), times when pain is horrible or excruciating (3).

For the present study, we created, three measures of pain by combining various items from the two existing CCRS RAI/MDS/FA 2.0 scales described above:

1. Pain-Yes/No (PAIN-Y/N): Pain prevalence dichotomous measure—no pain (0), pain (1) (mild, moderate, severe);
2. Four-point pain intensity scale (PI-4): no pain (0), mild pain (1), moderate pain (2), severe pain (3).

Research Objective and Data Analysis. The primary research objective was to examine the difference in pain between residents with and without hypertension. Inclusion criteria for the study incorporated all CCC residents with initial assessments from 2006–2016 reported in the CIHI CCRS dataset who were between the ages of 18 and 101 years and from Ontario-based CCC facilities. Non-parametric statistical tests, especially McNemar’s test for dichotomous pain and the Wilcoxon signed-rank test for ordinal pain, were used.

To evaluate the secondary research objective of exploring the effects of hypertension, sex, and hypertension according to sex interaction, binary and multinomial logistic regression analyses were used for PAIN-Y/N and for PI-4 outcomes, respectively. All regression models were conducted controlling for the following potential confounders: Activities of Daily Living (ADL) scores, Depression Rating Scale (DRS) scores, Index of Social Engagement (ISE) Scores, number of medications taken, days taking analgesics, and numbers of emergency room visits and physician visits).

All descriptive and inferential statistical analyses were performed in the software package SPSS (version 25, IBM). The level of statistical significance was used for inferential tests, with *p* < 0.05 (two-tailed) indicating statistically significant findings.

## 3. Results

The original dataset contained a total of 139,920 residents, of whom 77,323 (55.3%) had hypertension. Propensity matching with a 1:1 ratio was applied to the sample, resulting in matched pairs of 40,799 residents with hypertension and 40,799 residents without hypertension. Table 1 provides descriptive information about the matching criteria and control variables.

In comparing pain between residents with and without hypertension (Table 2), we observed a significantly lower proportion of hypertension residents with pain (71.7%) compared to controls (72.6%; McNemar’s test: *Χ*^2^ = 7.73, *p* = 0.005). There were significantly lower pain levels among cases compared to controls (*z* = 3.46, *p* = 0.001 for PI-4).

The proportion and severity of pain among females compared to males for both hypertension and control samples, respectively, are highlighted in Table 3 and Table 4.

There were 23,207 female (57%) and 17,592 male (43%) residents in each of the two groups. Table 2 and Table 3 show significantly higher pain (both proportion and severity) among females compared to males for both hypertension and control samples, respectively. Among both groups of residents, females had significantly higher odds of reporting pain in both pain measures (PAIN-Y/N, PI-4).

The associations of pain (PAIN-Y/N, PI-4) with hypertension, sex, and the interaction of hypertension and sex were evaluated (Table 5). For all models, the dependent variable was pain (with no pain being the reference category) and the independent variables were hypertension (coding: 0 for cases; 1 for controls), sex (coding: 0 for males; 1 for females), and the sex and hypertension interaction.

As shown in Table 5, examining the dichotomous pain outcome, significantly fewer residents with hypertension had pain than residents without hypertension (*OR* = 0.85 (95% CI 0.81–0.90), *p* < 0.001). Relative to males, females had 33% higher odds of reporting pain (*OR* = 1.33 (95% CI 1.26–1.40), *p* < 0.001). The hypertension and sex interaction effect was not significant. Further, residents with hypertension had lower odds of reporting all levels of pain in the PI-4: mild (*OR* = 0.88; 95% CI 0.83–0.94), moderate (*OR* = 0.86; 95% CI 0.81–0.91), and severe *(OR* = 0.69; 95% CI 0.63–0.76), all *p* < 0.001. When including sex as a predictor of pain, relative to males, females had 33% higher odds of reporting pain (model 1 *OR* = 1.33 (95% CI 1.26–1.40), *p* < 0.001). The hypertension and sex interaction effect was not significant (*p* = 0.315). Females reported higher pain levels than males for all categories of PI-4 (mild, moderate, and severe), with model 2 ORs being 1.31, 1.36, and 1.22, respectively, all *p* < 0.001. The significant interaction between hypertension and sex for severe pain (*OR* = 1.17 (95% CI 1.03–1.32), *p* = 0.014) and proportions from Table 6 show that non-hypertensive females had the highest proportion reporting severe pain (8.71%) compared to females with hypertension (8.03%) or males with and without hypertension (6.65% and 8.28%, respectively).

## 4. Discussion

The results of the present large-scale study indicate that the presence and severity of pain is significantly lower in complex chronic disease residents with a history of hypertension compared to a propensity-matched cohort without hypertension. Specifically, analysis showed that residents with hypertension had 15% lower odds of reporting pain than those without hypertension (*OR* = 0.85, model 1 in Table 5). These results were obtained even after adjusting for potential confounding variables, including psychological factors such as depression, activities of daily living and social engagement, and medical factors, including physician visits, emergency room visits, and medication and analgesic usage, which all may influence both hypertension and pain risks. The results support our hypothesis and the emerging evidence of a relationship between hypertension and reduced pain sensitivity [6,8,12,13,14,15,22,32,33,34], as well as building upon the complex chronic disease literature. Until now, despite significant health care concerns, the complex chronic disease population has remained unexplored in relation to hypertensive hypoalgesia

A number of viable physiological mechanisms have been offered as explanations of hypertensive hypoalgesia [7,18,35], including enhanced activation of baroreceptor reflex arcs, dysregulation of central endogenous opioid and noradrenergic activity, and increased stimulation of descending pain modulation pathways. As the central autonomic network includes a number of brain regions that are responsible for both cardiovascular regulation and pain modulation, one or more of these putative mechanisms of hypertensive hypoalgesia may be operating to reduce pain perception among individuals with high blood pressure and those at increased risk for the disorder.

Consistent with the existing pain literature [36,37,38,39,40], we found that females residents were 33% more likely to report pain compared to males (*OR* = 1.33, 95% CI 1.26–1.40, *p* < 0.001). A novel result was the finding of a significant interaction between hypertension and sex such that non-hypertensive females had the highest proportion reporting severe pain (8.71%) (Table 5 and Table 6). Sex differences in hypertensive analgesia research have been shown previously but in a different context. For example, France et al. (2009) evaluated the relationship between familial history of hypertension and neonatal pain in response to a vitamin K injection administered as routine care within the first 24 h after delivery. Pain responsiveness was measured using valid and reliable measures of facial grimacing and cry duration [41]. Neonates with a maternal family history of hypertension had significantly shorter crying times and marginally lower facial grimacing scores than neonates without a maternal or paternal history of hypertension during the vitamin K injection.

### 4.1. Strengths and Limitations

The results of the present study provide support for the concept of hypertensive hypoalgesia using a large sample of complex chronic disease residents. Although many factors contribute to pain intensity and frequency, the use of propensity matching to create demographically similar groups while also controlling for important psychological and medical variables allowed for a conservative comparison of pain outcomes among residents with and without hypertension. Propensity-matching statistics allow for a stringent comparison between groups, similar to a randomized controlled design [42].

Further, the benefits of examining secondary, standardized, electronic sources of longitudinal data arguably outweigh the limitations, particularly in epidemiological research. For instance, many of the challenges associated with primary data collection are avoided, including the time and resources that are required to hire research personnel and recruit participants [43]. A further strength of our study was the large, province-wide sample of CCD residents with and without hypertension in Ontario between the ages of 18 and 101 years, further contributing to the generalizability of the study. The large sample size of more than 40,000 residents per group also allowed for greater power in the analysis. The data utilized were of high quality, given the use of standardized electronic records throughout the CIHI Facility-Based Continuing Care Reporting System, which captures information on residents admitted to publicly funded hospital facilities for complex chronic care in Canada. The RAI/MDS/FA 2.0 utilized here is a validated tool, further contributing to the quality of the data [26].

Nonetheless, there are potential study limitations that should be kept in mind. Notably, the dataset utilized in this study did not include accessibility to more detailed resident histories, including body mass index (BMI), pain location and duration, and systolic, diastolic, and resting blood pressure readings. The consideration of these health, medication, and demographic factors may provide additional information about the hypertension and pain relationship. In this particular study, we did not examine the role of various therapies and treatments that are commonly applied for individuals with pain and/or hypertension (e.g., physiotherapy, psychotherapy, nutrition). This would be an interesting area to continue to explore, given the possible interaction of such treatments in the experience of pain and the management of hypertension. Finally, the information on pain status was based on self-reporting; however, this is a common limitation in pain research and relevant studies suggest that there is a moderate to strong agreement between self-reported disease status and medical records (National Center for Health Statistics, 1994).

### 4.2. Implications

This is the first study, to our knowledge, to explore the relation between hypertension and pain in a population of complex chronic disease residents. The results are consistent with previous research that supports the hypertensive hypoalgesia phenomenon, even after controlling for potential confounders. Given the complexity of the complex chronic disease population, information that may better inform the understanding of disease combinations and subsequent treatment care of this specific population is invaluable in an aging society.

## Figures and Tables

**Table 1:** Descriptive characteristics of matched samples.

Characteristics	Matched Hypertensive Patients (*n* = 40,799)	Control Group Patients	Comparison between Two Groups
		(*n* = 40,799)	
	N (%) or	N (%) or	
	Mean ± SD	Mean ± SD	
	Median (IQR)	Median (IQR)	
Gender			χ^2^(1) = 0.00, *p* = 1.00
Males	17,592 (43%)	17,592 (43%)	
Females	23,207 (57%)	23,207 (57%)	
Age	78.66 ± 10.54	78.66 ± 10.54	t(81,596) = 0.00,
			*p* = 1.00
Year of assessment			χ^2^(1) = 0.00, *p* = 1.00
2006	4045 (10%)	4045 (10%)	
2007	3889 (10%)	3889 (10%)	
2008	3696 (9%)	3696 (9%)	
2009	3810 (9%)	3810 (9%)	
2010	3998 (10%)	3998 (10%)	
2011	4399 (11%)	4399 (11%)	
2012	4170 (10%)	4170 (10%)	
2013	4069 (10%)	4069 (10%)	
2014	3861 (9%)	3861 (9%)	
2015	4028 (10%)	4028 (10%)	
2016	834 (2%)	834 (2%)	
Number of diseases (excluding hypertension)	1.65 ± 1.07	1.65 ± 1.07	χ^2^(6) = 0.00, *p* = 1.00
0			
1	5675 (14%)	5675 (14%)	
2	13,674 (33%)	13,674 (33%)	
3	12,929 (32%)	12,929 (32%)	
4	6578 (16%)	6578 (16%)	
5	1747 (4%)	1747 (4%)	
6	192 (1%)	192 (1%)	
	4 (0.01%)	4 (0.01%)	
Marital status			χ^2^(5) = 0.00, *p* = 1.00
Never married	2860 (7%)	2860 (7%)	
Married	18,483 (45%)	18,483 (45%)	
Widowed	15,491 (38%)	15,491 (38%)	
Separated	340 (1%)	340 (1%)	
Divorced	1505 (4%)	1505 (4%)	
Unknown	2120 (5%)	2120 (5%)	
Activities of Daily Living (ADL) score (long form)	16.36 ± 7.88	16.82 ± 8.30	t(81,596) = 8.08,
			*p* < 0.001
Depression Rating Scale (DRS) score	1.41 ± 2.11	1.42 ± 2.09	t(81,013) = 0.41,
			*p* = 0.68
Index of Social Engagement (ISE)	2.97 ± 2.04	2.72 ± 2.04	t(81,596) = 17.69,
			*p* < 0.001
Number of medications taken	12.73 ± 5.38	10.91 ± 5.18	t(81,596) = 49.04,
	Median (IQR):	Median (IQR):	*p* < 0.001
	12 (7)	10 (7)	M-W *p* < 0.001
Days taking analgesics	4.59 ± 3.04	3.56 ± 3.06	t(81,596) = 1.33,
	Median (IQR):	Median (IQR):	*p* = 0.19
	7 (6)	7 (6)	M-W *p* = 0.29
Number of emergency room visits	0.59 ± 0.90	0.59 ± 0.99	t(81,437) = 0.79,
	Median (IQR):	Median (IQR):	*p* = 0.43
	0 (1)	0 (1)	M-W *p* = 0.57
0	21,944 (54%)	22,111 (54%)	
1	15,607 (38%)	15,341 (38%)	
2	2165 (5%)	2226 (5%)	
4	584 (2%)	616 (2%)	
5 or more	407 (1%)	438 (1%)	
Number of physician visits	5.02 ± 3.30	5.15 ± 3.48	t(81,596) = 5.36,
	Median (IQR):	Median (IQR):	*p* < 0.001
	4 (5)	4 (5)	M-W *p* = 0.002
Hospital stays	1.14 ± 1.71	1.07 ± 1.27	t(81,437) = 6.86,
	Median (IQR):	Median (IQR):	*p* < 0.001
	1 (0)	1 (0)	M-W *p* < 0.001
0	8555 (21%)	9263 (23%)	
1	22,926 (56%)	23,214 (57%)	
2	6792 (17%)	6060 (15%)	
3	1722 (4%)	1598 (4%)	
4 or more	712 (2%)	597 (1%)	

Note: M-W is the Mann–Whitney non-parametric test.

**Table 2:** Comparison for dichotomous pain (PAIN-Y/N) and four-point pain scale (PI-4) between cases (hypertension) and controls (no hypertension), *n* = 40,799 in each group.

Pain Variable	Cases	Controls	Statistical
	N (%)	N (%)	Test
PAIN-Y/N			
No	11,522 (28.3%)	11,198 (27.4%)	McNemar’s test:
Yes	29,247 (71.7%)	29,601 (72.6%)	*Χ*^2^ = 7.73, *p* = 0.005
PI-4			
Mean ± SD *	1.28 ± 0.96	1.31 ± 0.97	
No pain (0)	11,522 (28.3%)	11,198 (27.4%)	Wilcoxon signed-ranks test: *z* = 3.46, *p* = 0.001
Mild pain (1)	9252 (22.7%)	9321 (22.8%)	
Moderate pain (2)	16,963 (41.6%)	16,801 (41.2%)	
Severe pain (3)	3032 (7.4%)	3479 (8.5%)	

* Mean and SD represent the mean and standard deviation of the rank-ordered pain scores.

**Table 3:** Comparisons for dichotomous pain (PAIN-Y/N) and four-point pain scale (PI-4) between males and females with hypertension (cases, *n* = 40,799).

Pain Variable	Females	Males	Statistical
	*n* = 23,207 (57%)	*n* = 17,592 (43%)	Comparison Test
PAIN-Y/N			
No	5653 (24.36%)	5899 (33.53%)	Chi-square test:
Yes	17,554 (75.64%)	11,693 (66.47%)	*Χ*^2^ = 415, *p* < 0.001
PI-4			
Mean ± SD	1.36 ± 0.94	1.18 ± 0.97	Mann–Whitney U test:
No pain (0)	5653 (24.36%)	5899 (33.53%)	*z* = 19.12, *p* < 0.001
Mild pain (1)	5390 (23.23%)	3862 (21.96%)	
Moderate pain (2)	10,301 (44.39%)	6662 (37.87%)	
Severe pain (3)	1863 (8.03%)	1169 (6.65%)	

**Table 4:** Comparisons for dichotomous pain (PAIN-Y/N) and four-point pain scale (PI-4) between males and females without hypertension (controls, *n* = 40,799).

Pain Variable	Females	Males	Statistical
	*n* = 23,207 (57%)	*n* = 17,592 (43%)	Comparison Test
PAIN-Y/N			Chi-square test:
No	5559 (23.96%)	5639 (32.05%)	*Χ*^2^ = 330, *p* < 0.001
Yes	17,648 (76.04%)	11,953 (67.95%)	
PI-4			
Mean ± SD	1.37 ± 0.94	1.22 ± 0.99	Mann–Whitney U test:
No pain (0)	5559 (23.96%)	5639 (32.05%)	*z* = 15.33, *p* < 0.001
Mild pain (1)	5473 (23.59%)	3848 (21.87%)	
Moderate pain (2)	10,153 (43.74%)	6648 (37.79%)	
Severe pain (3)	2022 (8.71%)	1457 (8.28%)	

**Table 5:** Binary and multinomial logistic regression models for dichotomous (PAIN-Y/N) and four-level (PI-4) variables. Each model has been adjusted to include Activities of Daily Living (ADL) scores, Depression Rating Scale (DRS) scores, Index of Social Engagement (ISE) scores, number of medications taken, days taking analgesics, and numbers of emergency room visits and physician visits.

Independent Variable	Dependent Variable	*B* (*SE*)	*p*-Value	*Odds Ratio* (95% CI)
Model 1: PAIN-Y/N				
Hypertension	Pain (Yes/No)	−0.159 (0.027)	<0.001	0.85 (0.81–0.90)
Sex	Pain (Yes/No)	0.285 (0.026)	<0.001	1.33 (1.26–1.40)
Hypertension × Sex	Pain (Yes/No)	0.037 (0.037)	0.315	1.04 (0.97–1.11)
Model 2: PI-4				
Hypertension	Mild pain (1)	−0.127 (0.031)	<0.001	0.88 (0.83–0.94)
	Moderate pain (2)	−0.149 (0.030)	<0.001	0.86 (0.81–0.91)
	Severe pain (3)	−0.374 (0.048)	<0.001	0.69 (0.63–0.76)
Sex	Mild pain (1)	0.273 (0.030)	<0.001	1.31 (1.24–1.39)
	Moderate pain (2)	0.308 (0.029)	<0.001	1.36 (1.29–1.44)
	Severe pain (3)	0.200 (0.044)	<0.001	1.22 (1.12–1.33)
Hypertension × Sex	Mild pain (1)	0.012 (0.042)	0.786	1.01 (0.93–1.10)
	Moderate pain (2)	0.038 (0.040)	0.350	1.04 (0.96–1.12)
	Severe pain (3)	0.156 (0.063)	0.014	1.17 (1.03–1.32)

**Table 6:** Percentages of male and female residents with and without hypertension reporting pain on the four-level pain scale (PI-4).

Pain Level	Male	Female
Pain (Yes/No)		
No hypertension	67.95%	76.04%
Hypertension	66.47%	75.64%
PI-4: Severe pain		
No hypertension	8.28%	8.71%
Hypertension	6.65%	8.03%
Odds ratio hypertension vs. no hypertension	0.77	0.91

## Data Availability

The original dataset was acquired through the Canadian Institute for Health Information Graduate Student Assistant Program (CIHI GDSAP).
